# Percutaneous left atrial appendage closure using the LAmbre device in patients with atrial fibrillation and left atrial appendage thrombus

**DOI:** 10.3389/fcvm.2022.1071119

**Published:** 2022-11-24

**Authors:** Lu-Shen Jin, Jin-Yan Ke, Yuan-Nan Lin, Ling Li, Jia-Yang Fu, Yi-Lian Chen, Yi-Xuan Qiu, Xiao-Wei Li, Yang-Qi Pan, Yue-Chun Li

**Affiliations:** Department of Cardiology, Second Affiliated Hospital and Yuying Children’s Hospital of Wenzhou Medical University, Wenzhou, China

**Keywords:** left atrial appendage closure, left atrial appendage thrombus, LAmbre device, left atrial appendage, atrial fibrillation

## Abstract

**Background:**

Left atrial appendage closure (LAAC) is considered a valid alternative for the prevention of thromboembolic stroke in patients with persistent left atrial appendage thrombus (LAAT) despite adequate anticoagulation. However, the data on LAAC using the LAmbre device for patients with LAAT is limited. This study was performed to explore efficacy and safety as well as to share the experience of the modified LAAC procedure with the LAmbre device.

**Materials and methods:**

A total of 7 patients with persistent LAAT despite adequate anticoagulation underwent modified LAAC with the LAmbre device between November 2019 and April 2022. Transesophageal echocardiography was performed 3 months postoperatively to detect device-related thrombosis and peridevice leak. The patients’ clinical events were evaluated during the perioperative and follow-up periods.

**Results:**

The median age, CHA_2_DS_2_-VASc score, and HAS-BLED score of all patients were 71 [53–73], 3 [2–4], and 2 [2–3], respectively. In the procedure, a cerebral protection system was used in two patients. LAAC with the LAmbre device was successfully performed in all patients without perioperative events. During the median follow-up of 383 [325–865] days, postoperative transesophageal echocardiography was performed in six (85.7%) patients. Device-related thrombosis was detected in one (16.7%) patient, and no significant peridevice leak was observed. No thromboembolic event or bleeding event occurred in any patients.

**Conclusion:**

LAAC with the LAmbre device is effective and safe when performed by experienced operators in highly selected patients with LAAT after adequate anticoagulation.

## Introduction

Atrial fibrillation (AF) is the most prevalent arrhythmia with an increased risk of atrial thrombosis, which forms most commonly in the left atrial appendage (LAA) in patients with non-valvular AF (1). Therefore, anticoagulation therapy is recommended for patients with AF to prevent stroke (2, 3). However, even with continuous anticoagulation, left atrial thrombus formation is still detected in a portion of patients (4–6). For these patients, intensification of antithrombotic therapy or surgical excision/occlusion of LAA was traditionally considered the only valid alternative treatment, as the presence of LAA thrombus (LAAT) was previously recognized as a contraindication to LAA closure (LAAC) (7, 8). Recently, several case reports have described the LAAC procedure in patients with persistent LAAT and suggested that it may be a valid alternative for these patients (9). However, most case reports focused on the Amplatzer cardiac plug and Amulet devices. As a widely used LAAC device, there is limited data on LAAC using the LAmbre (Lifetech Scientific Corporation, Shenzhen, China) device in patients with both AF and LAAT. The present study was performed to investigate the efficacy and safety of the modified LAAC procedure using the LAmbre device in patients with LAAT and to share our experience of this procedure.

## Materials and methods

### Study population

Seven patients with AF and LAAT who underwent modified LAAC with the LAmbre device at the Second Affiliated Hospital of Wenzhou Medical University from November 2019 to April 2022 were included in this study. Effective anticoagulation was administered for at least 3 months preceding the procedure. The patients’ baseline characteristics, CHA_2_DS_2_-VASc score, HAS-BLED score, anticoagulation medication, and transesophageal echocardiography (TEE) images were collected. The data collection and study were conducted according to the protocol approved by the Ethics Committee of the Second Affiliated Hospital of Wenzhou Medical University.

### The LAmbre device

The LAmbre is a self-expanding device consisting of an umbrella and a cover compliantly connected by a short central waist. The umbrella was specially designed for complete collapse and repositioning, which consists of 8 claws with separate stabilizing hooks for attachment to the LAA wall. The disk is larger than the umbrella, covers the LAA ostium, and sits alongside the chamber wall under mild tension. There are several sizes of umbrellas available, ranging from 16 to 36 mm in diameter, to accommodate variations of LAA anatomy.

### Procedure

**FIGURE 1:**
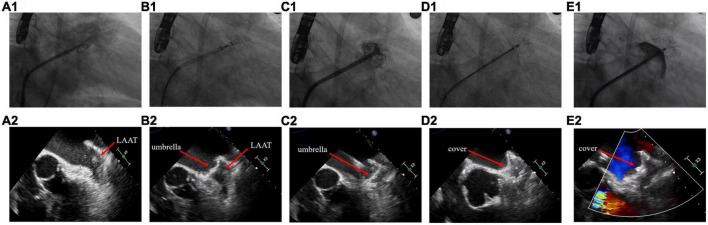
Left atrial appendage closure strategy in patients with left atrial appendage thrombus. **(A)** Fluoroscopy and transesophageal echocardiography images before the procedure. **(B)** The umbrella of the LAmbre device was partially deployed at the ostium of left atrial appendage (LAA). **(C)** The umbrella was propelled distally to the LAA and expanded. **(D)** The cover was deployed to seal the LAA ostium. **(E)** Detection of peridevice leak.

The procedures were performed under general anesthesia or local anesthesia. TEE was performed preoperatively to determine the location of the thrombus in relation to the LAA and the diameter of the device landing zone and LAA ostium. If any part of the thrombus was located between the landing zone and the LAA ostium, it was considered a proximal LAAT; otherwise, it was considered a distal LAAT. The choice of the device size and whether to use the cerebral protection system (Emboshield NAV^6^; Abbott Laboratories, Abbott Park, IL, USA) were at the discretion of the operator based on the TEE images. If the cerebral protection system was absent, a neurologic specialist would prepare for interventional treatment during the entire procedure. The procedure steps are as follows: (1) Transseptal access was obtained via the right femoral vein under fluoroscopic guidance, and a guidewire was slowly advanced into the left atrium. (2) The operator turned the Swartz sheath clockwise to facilitate the advancement of the guidewire into the left superior pulmonary vein. The guidewire must be manipulated carefully to avoid insertion of the LAA and contact with the LAAT. (3) The operator replaced the Swartz sheath with a delivery sheath and then delivered the pigtail catheter in the left superior pulmonary vein. (4) The operator dropped the pigtail catheter to the LAA ostium by slowly pulling the sheath and catheter back. Unlike the conventional LAAC procedure, the operator must keep the pigtail catheter in the sheath opening during this step to avoid random movement leading to thrombus dislodgement. (5) If the locational relationship between the pigtail catheter and the thrombus was appropriate, a small amount of contrast was injected to confirm the LAA anatomy. Intraoperative TEE was performed to reconfirm the location of the LAAT and accurately measure the LAA ostium and landing zone diameters for precise selection of the device size, which is critical to achieve a one-shot release. (6) The umbrella of the LAmbre device was initially two-thirds deployed at the ostium of LAA, and the whole system was then propelled under counterclockwise rotation to lock up the thrombus by pushing it to the distal part of the LAA. After advancement to the landing zone, the device subsequently completed deployment. The sheath was withdrawn to expand the proximal cover. (7) TEE, fluoroscopy, and a sustained tug test were then performed to confirm the proper position of the device and complete occlusion of the LAA. Finally, the device was released (Figure 1 and Supplementary Videos 1–12).

### Anticoagulation and follow-up

After the procedure, the patients received rivaroxaban or warfarin for 3 months based on the oral anticoagulants taken preoperatively. If TEE showed satisfactory positioning of the LAmbre device, a peridevice leak of <3 mm, and no device-related thrombosis (DRT) at 3 months postoperatively, oral anticoagulants would be replaced by clopidogrel in the long term. For patients with DRT, anticoagulants were continued until follow-up TEE detected the dissolution of DRT. The patients were evaluated for the development of transient ischemic attack, stroke, systemic embolism, and major bleeding events throughout follow-up. These events were defined according to the Munich consensus document (10).

### Statistics

Statistics analysis was performed with SPSS software, version 26.0 (IBM Corp., Armonk, NY). Numbers are shown as median [interquartile range].

## Results

### Patient characteristics

**FIGURE 2:**
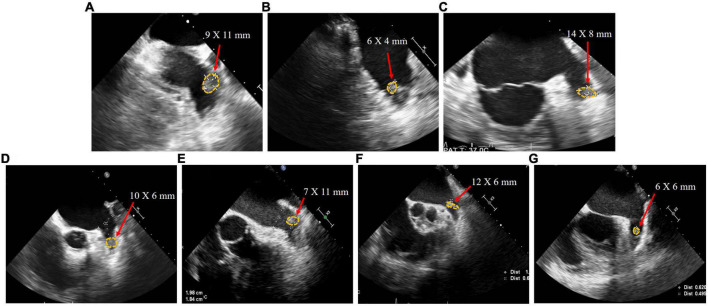
Transesophageal echocardiography images of patient **(A–G)** with left atrial appendage thrombus before left atrial appendage closure.

Seven patients (Patients A–G) were included in this study. Their baseline characteristics are shown in Table 1. The median age, CHA_2_DS_2_-VASc score, and HAS-BLED score of patients were 71 [53–73], 3 [2–4], and 2 [2–3], respectively. Patient B had paroxysmal AF, whereas all other patients had persistent AF. All patients who presented with persistent LAAT had received adequate anticoagulation therapy for at least 3 months after thrombus detection, involving warfarin (international normalized ratio 2.0–3.0) and standard doses of rivaroxaban according to creatinine clearance. The thrombus in Patients A, E, F, and G was located proximal to the LAA, whereas the thrombus in the other patients was located distal to the LAA (Figure 2).

**TABLE 1:** Patient characteristics.

Patient	A	B	C	D	E	F	G
Male	Yes	Yes	Yes	Yes	Yes	Yes	Yes
Age	72	47	68	71	73	74	53
CHA_2_DS_2_-VASc	4	4	4	2	3	2	2
HAS-BLED	2	3	4	3	2	2	1
Hypertension	No	Yes	Yes	Yes	No	Yes	Yes
Diabetes mellitus	No	Yes	No	No	No	No	No
LVEF	66	72	62	63	65	68	30
Liver disease	No	No	No	No	No	No	No
Vascular Diseases	Yes	No	No	No	No	No	No
Bleeding history	No	No	No	No	No	No	No
Prior stroke/TIA	Yes	Yes	Yes	No	Yes	No	No
AF type	Persistent	Paroxysmal	Persistent	Persistent	Persistent	Persistent	Persistent
Anticoagulants	1.875 mg warfarin	20 mg rivaroxaban	20 mg rivaroxaban	2.5 mg warfarin	2.5 mg warfarin	15 mg rivaroxaban	20 mg rivaroxaban
Location of LAAT	The proximal of LAA	The distal of LAA	The distal of LAA	The distal of LAA	The proximal of LAA	The proximal of LAA	The proximal of LAA
LAA morphology	Cauliflower	Cauliflower	Cauliflower	Chicken wing	Cauliflower	Cauliflower	Cauliflower

AF, atrial fibrillation; LAAT, left atrial appendage thrombus; LVEF, left ventricular ejection fraction; TIA, transient ischemic attacks.

### Procedural outcomes

**FIGURE 3:**
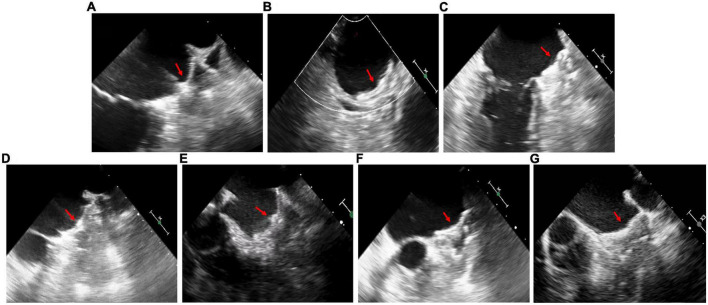
Transesophageal echocardiography images in patient **(A–G)** after left atrial appendage closure. The red arrows point to devices.

Patients D and E underwent general anesthesia due to intolerance of TEE, while the other patients received local anesthesia. The cerebral protection system was only used in Patients A and E, in which case no thrombus was collected. The LAmbre device was successfully implanted (Figure 3) in the first attempt in all patients except Patient B (Table 2). During the procedure in Patient B of “cauliflower” LAA, the partially deployed device did not successfully reach the landing zone and showed significant displacement after deployment. As the operator retracted the device slowly with TEE monitoring the state of the thrombus, the LAmbre device was completely retrieved without thrombus dislodgement. The operator then selected the upper lobe as the landing zone and advanced the catheter close to the superior edge of the LAA to avoid touching the thrombus. Finally, the LAmbre device was successfully released to complete LAAC. The median length of stay for all patients was 2 days. No major bleeding, pericardial effusion, or embolization events were observed during the periprocedural period (Table 3).

**TABLE 2:** Procedural characteristics.

Patient	A	B	C	D	E	F	G
Anesthesia protocol	Local	Local	Local	General	General	Local	Local
Device size	30/36 mm	16/30 mm	22/28 mm	26/32 mm	30/36 mm	24/30 mm	28/34 mm
Device deployed on the first attempt	Yes	No	Yes	Yes	Yes	Yes	Yes
Cerebral protection device use	Yes	No	No	No	Yes	No	No
Procedural success	Yes	Yes	Yes	Yes	Yes	Yes	Yes

**TABLE 3:** Periprocedural outcomes.

Patient	A	B	C	D	E	F	G
In-hospital days after procedure	3	2	3	2	2	2	3
Anticoagulants	1.875 mg warfarin	20 mg rivaroxaban	20 mg rivaroxaban	2.5 mg warfarin	2.5 mg warfarin	15 mg rivaroxaban	20 mg rivaroxaban
Death	No	No	No	No	No	No	No
Bleeding	No	No	No	No	No	No	No
Embolization	No	No	No	No	No	No	No
Pericardical effusion	No	No	No	No	No	No	No
Vascular complications	No	No	No	No	No	No	No
Peridevice leak	≤2 mm	No	No	No	No	No	No

### Follow-up

**FIGURE 4:**
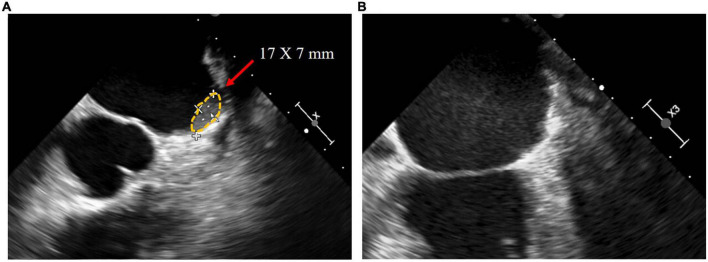
In the transesophageal echocardiography images of patient B, **(A)** DRT of 17*7 mm was detected 14 months postoperatively, and **(B)** DRT dissolved after 5 months of 2.5 mg warfarin once daily.

The median follow-up duration was 383 [325–865] days. The anticoagulation regimen for patients after the procedure is shown in Table 3. TEE was performed 3 months after the procedure in all patients except Patient D, who refused TEE because of intolerance. The peridevice leaks of ≤ 2 mm were found in Patients A, B, and E, and no DRT was detected. At 14 months postoperatively, Patient B underwent a repeat TEE examination because of discontinuation of clopidogrel for 2 months without authorization, and a DRT (17 × 7 mm) was found. The physician changed Patient B’s regimen to 2.5 mg warfarin once daily to dissolve the DRT. After 5 months on warfarin, TEE reexamination showed complete dissolution of the DRT (Figure 4). During follow-up, no bleeding event, cerebrovascular event, or death occurred in any patients (Table 4).

**TABLE 4:** Follow-up outcomes.

Patient	A	B	C	D	E	F	G
Follow-up duration (days)	927	865	851	383	325	376	182
Death	No	No	No	No	No	No	No
Stroke or TIA	No	No	No	No	No	No	No
DRT	No	Yes	No	–	No	No	No
bleeding	No	No	No	No	No	No	No
embolization	No	No	No	No	No	No	No
Pericardical effusion	Yes	No	No	No	No	No	No
Peridevice leak	≤2 mm	≤2 mm	No	–	≤2 mm	No	No

DRT, device-related thrombus; TIA, transient ischemic attacks.

## Discussion

This study explored the effect of the modified LAAC procedure with the LAmbre device in patients with persistent thrombosis despite long-term anticoagulation therapy. Successful LAAC was performed without thrombus dislodgement in all patients of this study. No clinical complications occurred during the periprocedural or follow-up period, although DRT was confirmed in one patient.

### Left atrial appendage closure with left atrial appendage thrombus

Previous studies have shown that in the presence of adequate anticoagulation, the thrombus dissolved in only 76–79% of patients with LAAT (11, 12). For these patients with persistent LAAT and a high risk of embolic events, invasive approaches such as surgical removal of LAAT with LAA excision have been recommended to prevent stroke. However, the drawbacks of open surgery are obvious, including serious trauma, high adverse event rates, and a longer time to recover, particularly unbearable for elderly patients. In this scenario, LAAC may be a better option as a minimally invasive procedure. Several case reports have recently confirmed the effectiveness of LAAC in patients with LAAT (9). Nevertheless, the data on LAAC using the LAmbre device for patients with LAAT is limited. To the best of our knowledge, the present study is the largest study to explore the efficacy and safety of the modified LAAC procedure with the LAmbre device in patients with LAAT, as well as to share the experience of the procedure.

### Advantages of the LAmbre device

The LAmbre device is divided into a distal umbrella and a proximal cover, which is designed for proximal implantation. The umbrella of the device can be partially deployed at the ostium of the appendage and pushed inward to prevent embolism by propelling the thrombus to the distal LAA. During the operation, the delivery sheath does not reach the distal LAA position, thus effectively reducing the possibility of contact with the thrombus. In contrast, the Watchman device, a widely used conventional “plug” device, requires the delivery sheath to reach more deeply into the LAA during the procedure, potentially leading to thrombus dislodgement. Other “disk” devices, such as the Amplatzer cardiac plug and Amulet device, also allow this operation. However, the LAmbre device is available in a variety of sizes and adjustable sheaths to accommodate various LAA morphologies, especially shallow or multilobular LAA anatomies (13, 14).

### Characteristics of the procedure

In patients with thrombosis undergoing LAAC, the site and size of the thrombus are associated with the risk of the procedure. A thrombus close to the apex of the LAA is less likely to be contacted, implying a lower risk of thrombus dislodgement. For a proximal thrombus in the LAA, the procedure also has a relatively high success rate and safety if the thrombus is small and easy to push distally. However, some thrombi, such as large thrombi overhanging the LAA and long strips of thrombi attached to the LAA are not suitable for LAAC because it cannot be safely and effectively pushed to the distal part of the LAA. Therefore, in previous case reports of LAAC for the treatment of LAAT, the majority of patients had thrombi located distal to the LAA (9). A few studies of LACC included patients with proximal thrombi, whereas patients with thrombi overhanging the ostium were often excluded (15–18). In the present study, the thrombus in four of seven patients with LAAT was located proximal to the LAA, which is associated with a high risk of thromboembolic events. As a result of a detailed preoperative evaluation and an appropriate procedure performed by experienced operators, none of the patients in this study developed thrombus dislodgement or other complications.

Most of the patients in our study received local anesthesia. Under local anesthesia, the operator and the neurologist can stay informed of the patient’s neurological condition and take prompt action in case of cerebral embolism. Therefore, local anesthesia is recommended for the modified LAAC procedure unless the patient cannot tolerate TEE.

During the LAAC procedure, manipulation within the LAA must be minimized to ensure safety. In some case reports of LAAC for treatment of LAAT, contrast injection, which was used for measurement of LAA size and morphology in the conventional LAAC procedure (19), was often omitted to prevent thrombus dislodgement. The operators in these reports performed the implantation based on TEE imaging (17, 20, 21). In other reports, the operators took the approach of injecting a small amount of contrast with the pigtail catheter placed shallowly into the LAA (18, 22, 23). In the present study, the operator dropped the pigtail catheter to the LAA ostium by withdrawing it after having replaced it in the left upper pulmonary vein. A small amount of contrast was injected if the locational relationship between the thrombus and pigtail catheter was appropriate. No embolism or stroke event occurred throughout the perioperative period. The moderate utilization of small amounts of contrast contributed to the success of the modified LAAC procedure without leading to intraoperative embolization.

For patients with thrombi, a one-shot release is desired to avoid complications because there is an increased risk of thrombus dislodgement during device repositioning or replacement. In Patient B of the present study, the device was confirmed unstable by a sustained tug test during the procedure. The operator had to replace the device for a successful LAAC. After retracting the cover and returning the umbrella to a partially open state, the operator confirmed no thrombus displacement and then gradually retracted the umbrella under TEE monitoring. This case provides a reference for LAAC when the thrombus impairs the stability of device. Notably, however, our operator had extensive experience in performing LAAC, and this procedure had a high risk of thromboembolic events.

In this study, a cerebral protection system consisting of a filter composed of 120 μm micropores and a filter delivery wire was used in Patients A and E. The operator inserted 6F sheaths and deployed two filters into both internal carotid arteries through the femoral arteries to prevent devastating stroke complications. Previous studies have demonstrated the effectiveness of the cerebral protection system for periprocedural stroke prevention in patients with LAAT (16, 24, 25). However, despite the implantation of such a protection system, cerebral ischemic events still cannot be completely eliminated because the vertebral artery is unprotected. Embolic events can also occur in peripheral arteries, such as lower extremity arteries, mesenteric arteries, and renal arteries. In addition, prolongation of the operation and additional device manipulation in the carotid artery increase the risk of perioperative complications. The extra financial burden limits the use of such devices in selected patients. Indeed, because the utilization of a cerebral protection system depends on the operators’ experience and skill, there is no standard to be followed. The operators in this study considered it appropriate to deploy the cerebral protection system in patients with LAAT close to the ostium because these patients were prone to thrombus dislodgement. It must be emphasized that if a cerebral protection system is unavailable, a neurologic specialist must be prepared for interventional treatment during the entire procedure.

### Follow-up

In our study, no thromboembolic or bleeding events occurred in any patient during the median follow-up of 383 [325–865] days. According to the HAS-BLED score and CHA_2_DS_2_-VASc score, the incidence of major bleeding events and the incidence of thromboembolic events were both reduced by 100% in our study compared with the expected annual rate (26). However, the risk of thromboembolic events in our study might be higher than that in previous studies because our patients had persistent thrombi despite anticoagulation therapy.

In the postoperative TEE screenings of six patients, DRT was detected in the second TEE screening of Patient B at 14 months after the procedure. This may be due to his discontinuation of clopidogrel. After treatment with warfarin for 5 months, the repeat TEE confirmed the dissolution of the DRT, and no clinical events occurred. These results reflect the efficacy and safety of LAAC in patients with LAAT. However, the long-term effect and safety require confirmation.

### Limitations

This study has three main limitations. First, it was a single-center retrospective study with a small sample size. The results are not generalizable because the patients who underwent the modified procedure were highly selected and the operators were experienced. Second, patients with silent brain ischemia and subclinical microembolization could not be excluded because brain magnetic resonance imaging and neurologic assessment were not performed. Thirdly, the anticoagulation therapy after LAAC was not identical in patients, which may affect the incidence of DRT. Finally, the risk of long-term complications is unclear due to a relatively short follow-up duration.

## Conclusion

This study has shown that the modified LAAC procedure with the LAmbre device is safe and effective in selected patients with LAAT when performed by experienced operators. This procedure might be an option for patients with AF and LAAT in whom oral anticoagulants are ineffective. However, large randomized studies with long-term follow-up are required to confirm the safety and efficacy.

## Data availability statement

The original contributions presented in this study are included in the article/Supplementary material, further inquiries can be directed to the corresponding author.

## Ethics statement

The studies involving human participants were reviewed and approved by the Ethics Committee of the Second Affiliated Hospital of Wenzhou Medical University. The patients/participants provided their written informed consent to participate in this study.

## Author contributions

L-SJ, Y-NL, and Y-CL contributed to the conception and design of the study. Y-QP and X-WL organized the database and performed the statistical analysis. L-SJ, J-YK, and Y-NL wrote the first draft of the manuscript. LL, J-YF, Y-LC, and Y-XQ wrote sections of the manuscript. All authors contributed to the manuscript revision, read, and approved the submitted version.
